# Type 2 Diabetes and Causes of Sudden Cardiac Death: A Systematic Review

**DOI:** 10.7759/cureus.18145

**Published:** 2021-09-20

**Authors:** Karan B Singh, Maduka C Nnadozie, Muhammad Abdal, Niki Shrestha, Rose Anne M Abe, Anum Masroor, Arseni Khorochkov, Jose Prieto, Lubna Mohammed

**Affiliations:** 1 Internal Medicine, California Institute of Behavioral Neurosciences & Psychology, Fairfield, USA; 2 Emergency Medicine, California Institute of Behavioral Neurosciences & Psychology, Fairfield, USA; 3 Research, California Institute of Behavioral Neurosciences & Psychology, Fairfield, USA; 4 Psychiatry, California Institute of Behavioral Neurosciences & Psychology, Fairfield, USA; 5 Psychiatry, Psychiatric Care Associates, Englewood, USA; 6 Medicine, Khyber Medical College, Peshawar, PAK

**Keywords:** coronary artery disease (cad), diabetes type 2, sudden cardiac arrest, hypoglycemia, autonomic nervous system dysfunction, increased blood sugar levels, life threatening arrhythmia, atherosclerosis, chronic kidney disease (ckd), electrolyte imbalance

## Abstract

Type 2 diabetes mellitus has been on the rise in recent years. A major cause of death in the United States is myocardial infarction with underlying coronary artery disease. Impairment of tissue insulin sensitivity in type 2 diabetes is a significant factor for sudden cardiac death. The complex pathophysiology stems from coexisting cardiovascular disease and complications of impaired tissue sensitivity to insulin. Long-term diabetics with underlying kidney disease and those requiring dialysis have systemic inflammation that adds to an increased risk of death. During times of pathological stress, myocardial tissue will express substrates and growth factors that cause conduction disequilibrium and predispose to sudden cardiac death. Diabetes is a modifiable risk factor in the prevention of sudden cardiac arrest. Specific prevention measures aimed towards lifestyle modification and medications are important to prevent diabetes and decrease mortality of future cardiac death. In recent times, drugs that compete with glucose in the proximal convoluted tubule of the nephron have clinical significance in lowering the risk of sudden cardiac arrest.

## Introduction and background

A projection into 2025 indicates that more than 380 million individuals will have type 2 diabetes [1]. Although the incidence of type 2 diabetes is on the rise, the complications of acute myocardial infarction and cerebrovascular accidents are decreasing [1]. The annual deaths due to immediate cardiac arrest in the USA are 180,000 to 450,000 deaths per year [2-4]. Cardiac complications are mainly due to coronary heart disease, the most common risk factor for sudden cardiac death (SCD) in diabetes, affecting ~80% of males and ~45% of females [2]. Factors that increase cardiovascular diseases such as hypertension, hyperlipidemia, and obesity are substantially higher in patients with diabetes. These patients have pre-existing hypertension in 76% of the cases and coronary heart disease in 36% of cases [5]. In approximately 50% of individuals, the insult leading to sudden death with coronary artery disease is underlying atherosclerotic changes [2]. Presumably, the underlying cause of death is an alteration in the heart’s electrical circuit precipitating ventricular tachycardia in approximately 63% of individuals [2].

Diabetes can be categorized into three subtypes: Type 1 diabetes mellitus/insulin-dependent diabetes mellitus (T1DM/IDDM), type 2 diabetes/non-insulin-dependent diabetes mellitus (T2DM/NIDDM), and gestational diabetes mellitus (GDM). These conditions have impaired glucose uptake from the bloodstream as a common theme. The pancreas secretes hormones that maintain euglycemia, and insulin is responsible for lowering blood glucose levels. In T1DM, there is a decreased release of insulin from the beta-pancreatic cells. On the other hand, in T2DM, there is impaired insulin sensitivity because of defective insulin receptors on the cell membrane. In medical terminology, hyperglycemia means elevated serum glucose levels. Over time, hyperglycemia leads to non-enzymatic glycosylation of proteins, especially hemoglobin. This process leads to the formation of hemoglobin A1c (HbA1c). A fasting blood sugar of >126 mg/dL or HbA1c of >6.5% or a two-hour postprandial blood sugar level of >200 mg/dL is the objective definition of diabetes mellitus [6]. T1DM essentially requires insulin for its management; whereas, T2DM may also require insulin at some stage in many patients [7]. Diabetes serves as a substrate, targeting mainly the cardiovascular system and causing coronary heart disease, stroke, peripheral arterial disease, and autonomic neuropathy. The management of T2DM is targeted to strict glycemic, blood pressure, and cholesterol control, as these interventions decrease the risk of cardiovascular disease by ~50% [8].

A sudden death of cardiac etiology has many risk factors such as coronary heart disease, dilated cardiomyopathy, age, and sex. In the middle-aged group, 45 to 65 years old, the most common cause of sudden cardiac arrest is underlying coronary heart disease [9]. Generally, elderly males are more likely to have this condition than their female counterparts [2]. There are approximately 300,000 deaths suddenly due to cardiac reasons in the United States per year; however, in recent years, this trend is declining [3]. Patients undergoing hemodialysis die commonly due to underlying cardiac disease [10]. The decrease in sudden deaths is related to the interventions geared towards the risk factors and healthy lifestyle maintenance that improve diseases like diabetes, hypertension, and hyperlipidemia [2,11]. Although sudden cardiac demise is multifactorial, about 40% of deaths are unknown [12]. Prevention of sudden arrest is preventable with a prophylactic implantable cardioverter-defibrillator (ICD) in select patients [13-15].

The main objective of this systematic review is to elucidate different contributing variables that are associated with and predispose T2DM to sudden cardiac death.

Methods

For this systematic review, the Preferred Reporting Items for Systematic Review and Meta-Analyses (PRISMA) 2009 Guidelines were used for reference. The articles are searched from PubMed, PubMed Central, and Medline using keywords. The following keywords were used in the search strategy: Type 2 Diabetes OR Non-insulin-dependent diabetes mellitus OR Hypoglycemia AND Sudden cardiac death OR Silent ischemia. In addition, the National Library of Medicine database was incorporated using the medical subject headings (MeSH) technique. The finalized search strategy is a combination of keywords and MeSH using Booleans: Type 2 Diabetes OR Non-insulin-dependent diabetes mellitus OR Hypoglycemia OR "Diabetes Mellitus, Type 2/blood"[Majr] OR "Diabetes Mellitus, Type 2/complications"[Majr] OR "Diabetes Mellitus, Type 2/diagnosis"[Majr] OR "Diabetes Mellitus, Type 2/diet therapy"[Majr] OR "Diabetes Mellitus, Type 2/drug therapy"[Majr] OR "Diabetes Mellitus, Type 2/mortality"[Majr] OR "Diabetes Mellitus, Type 2/pathology"[Majr] OR "Diabetes Mellitus, Type 2/physiology"[Majr] OR "Diabetes Mellitus, Type 2/physiopathology"[Majr] OR "Diabetes Mellitus, Type 2/prevention and control"[Majr] AND Sudden cardiac death OR Silent ischemia OR "Death, Sudden, Cardiac/etiology"[Majr] OR "Death, Sudden, Cardiac/pathology"[Majr] OR "Death, Sudden, Cardiac/prevention and control"[Majr]. The search strategy and keywords are mentioned below (Tables 1, 2). 

**Table 1:** MeSH strategy combined with keywords

Combined MeSH and Keywords search	Database	Number of Results	Inclusion/Exclusion	Duplicates removed
Type 2 Diabetes OR Non-insulin dependent diabetes mellitus OR Hypoglycemia OR "Diabetes Mellitus, Type 2/blood"[Majr] OR "Diabetes Mellitus, Type 2/complications"[Majr] OR "Diabetes Mellitus, Type 2/diagnosis"[Majr] OR "Diabetes Mellitus, Type 2/diet therapy"[Majr] OR "Diabetes Mellitus, Type 2/drug therapy"[Majr] OR "Diabetes Mellitus, Type 2/mortality"[Majr] OR "Diabetes Mellitus, Type 2/pathology"[Majr] OR "Diabetes Mellitus, Type 2/physiology"[Majr] OR "Diabetes Mellitus, Type 2/physiopathology"[Majr] OR "Diabetes Mellitus, Type 2/prevention and control"[Majr] AND Sudden cardiac death OR Silent ischemia OR "Death, Sudden, Cardiac/etiology"[Majr] OR "Death, Sudden, Cardiac/pathology"[Majr] OR "Death, Sudden, Cardiac/prevention and control"[Majr]	PubMed.	12,239 results.	791 results.	187 duplicates removed.

**Table 2:** Main Keywords

Key Word	Database	Number of Results	Inclusion/exclusion
Type 2 Diabetes OR Non-insulin dependent diabetes mellitus OR Hypoglycemia AND Sudden cardiac death OR Silent ischemia	PubMed.	4,669 results.	280 results.

Eligibility

The articles are screened for eligibility using inclusion/exclusion criteria. Papers from PubMed included the following: English language, a population of middle-aged and aged 45+ years, publications within the last 10 years, free, full-text articles, and only human participants. All young patients that died suddenly from extracardiac etiologies and neurological causes were excluded from the study.

Results

**Figure 1:**
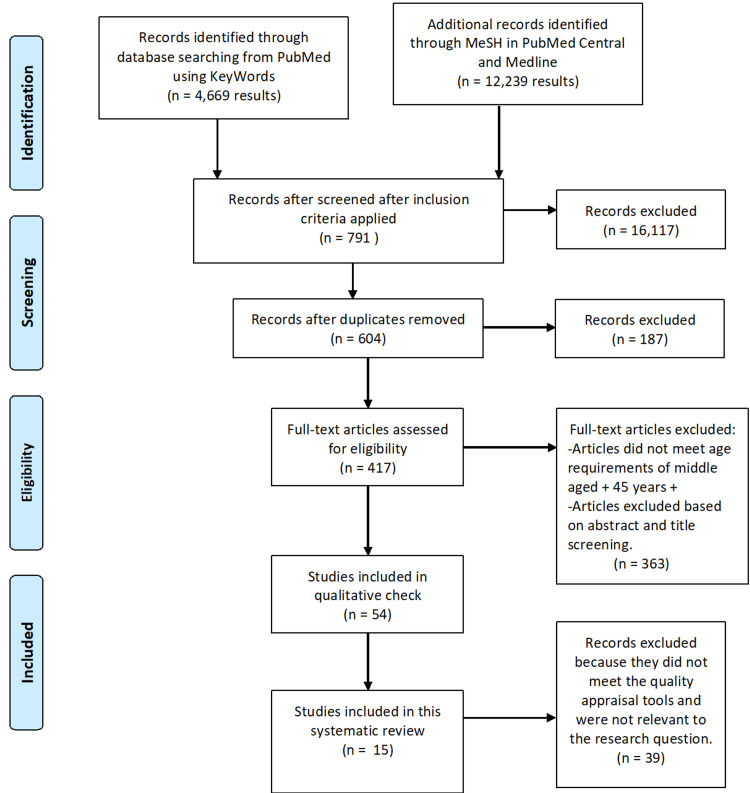
PRISMA (2009) flow diagram for the present study. PRISMA-Preferred Reporting Items for Systematic Reviews and Meta-Analyses.

A total of 16,908 articles were screened from the PubMed, PubMed Central, and Medline databases. From this, 16,304 articles were filtered based on the inclusion/exclusion criteria, and duplicates were removed with EndNote. The remaining 417 papers were evaluated for title and abstract. During the initial screening, 54 selected articles met the eligibility criteria. In the end, this study includes 15 full-text articles after the use of quality assessment tools. Of the 15 studies, nine are cohorts, two are randomized control trials (RCTs), two are observational studies, one cross-sectional study, and a review article. The Cochrane risk-of-bias tool assessed the RCTs. The review article and observational studies utilized the Scale for the Assessment of Narrative Review Articles (SANRA) checklist and the Newcastle Ottawa Scale for excellent quality appraisal, respectively. The final articles included were independently checked by another author (Nnadozie MC). The summarized studies for this systematic review are included in Table 3 and the PRISMA flowchart in Figure 1 given below.

**Table 3:** Results of the studies included (n = 15) AF-Atrial Fibrillation. BMI-Body Mass Index. HbA1c-Hemoglobin A1c. QTc-QT corrected. HCM-Hypertrophic Cardiomyopathy. T2DM-Type 2 Diabetes Mellitus. SCD-Sudden Cardiac Death. N/A-Not Available.

Study		Author	Year	Type of Study	Patients	Purpose of Study	Results	Conclusion
1.	Wang et al. [16].		2020	Cohort study.	67	T2DM impact on death in patients with hypertrophic cardiomyopathy.	There is a higher rate of death within three years in hypertrophic cardiomyopathy with diabetes vs without.	Diabetes has a direct correlation with death in the first three years after ablation procedure in HCM. There are other risk factors that have independent effects as well.
2.	Leonard et al. [17].		2020	Cohort study.	Medicaid patients with T2DM.	Comparing the cases of arrhythmias and sudden death in patients taking rosiglitazone or pioglitazone.	Taking these diabetic medications has the risks of sudden cardiac arrest and malignant arrhythmias.	Both drugs have similar effects on outcomes for death.
3.	Fitchett et al. [18].		2019	Randomized control trial.	7,020	Effects of empagliflozin in type 2 diabetics with cardiovascular disease.	All patients that received the drug had a decreased mortality and cardiovascular outcome regardless of group characteristics.	Diabetics with the preexisting cardiovascular disease still benefit from pharmacological therapy with empagliflozin.
4.	Prasad et al. [19].		2019	Observational study.	338	To determine the risk factors and prevalence of silent cardiac injury in asymptomatic non-insulin-dependent diabetes mellitus. This allows detection of atherosclerosis earlier.	Patients with positive screening tools for underlying atherosclerosis were mostly diabetic males >50 years old with other risk factors for coronary artery disease.	Approximately every one in four clinically asymptomatic type 2 diabetics have silent cardiac ischemia.
5.	Kobayashi et al. [20].		2018	Cross-sectional study.	219	QT prolongation and the role of sudden cardiac death in type 2 diabetics.	There is an increased risk of prolonged QTc in patients with prolonged diabetes, female sex, insulin therapy, BMI, and systolic pressure.	As the microvascular complications progress, there is an increased risk of sudden cardiac death with prolonged QTc.
6.	Weidner et al. [6].		2018	Cohort study.	2,411	T2DM and the significance of fatal arrhythmias presenting in hospital admissions.	Diabetes when present has a higher all-cause mortality rate for cardiac arrhythmias.	The presence of non-insulin-dependent diabetes mellitus was an independent variable for survival in patients presenting with arrhythmias.
7.	Chao et al. [21].		2017	Cohort study.	>23 million	Whether AF increases the risk of sudden cardiac death.	AF along with other risk factors such as diabetes are associated with sudden cardiac death.	The risk of sudden cardiac arrest in AF was 64% higher than in non-AF patients.
8.	Charytan et al [22].		2016	Observational study.	66	Investigate incidence of sudden cardiac death in dialyzed patients.	Most individuals undergoing dialysis have underlying cardiovascular disease that predisposes them to cardiac sentinel events including sudden death.	During the dialysis cycle, patients are at an increased risk for sudden cardiac death, and placing implantable cardiac monitoring will allow immediate adjustments in the dialyzer.
9.	Eranti et al. [7].		2016	Cohort study.	10,594	Compare whether cardiac death is sudden in diabetic patients vs non-diabetic patients.	Impairment of glucose utilization subjected patients to be at higher risk for sudden cardiac death.	Diabetes increases the risk of arterial disease and the likelihood of immediate cardiac arrest.
10.	Hempe et al. [23].		2015	Randomized control trial.	10,251	Diabetics that are randomized and treated intensively produce a higher rate of adverse clinical outcomes.	Individuals in the higher hemoglobin glycation index had increased rates of hypoglycemia and mortality	Decreasing HbA1c to below 6% produced increased cardiovascular outcomes such as death.
11.	Chitnis et al. [24].		2014	Cohort study.	929	Determination of predictors for sudden cardiac death in patients with underlying heart disease.	Patients with a lower ejection fraction had a higher prevalence of diabetes.	The group with higher individuals with diabetes had a higher association with sudden cardiac death.
12.	Davis et al. [25].		2013	Cohort study.	5,102	Prevalence of silent cardiac injury in newly diagnosed T2DM and its correlation with future mortality.	Patients that had silent myocardial infarction had a higher hazard ratio for fatal outcomes and increased mortality.	20% of newly diagnosed diabetics are at risk for silent infarctions of the heart.
13.	Snell-Bergeon et al. [26].		2012	Traditional review.	N/A	Effects of hypoglycemia and cardiovascular outcomes in diabetics.	Hypoglycemia increases the risk of cardiovascular outcomes such as death and arrhythmias in the acute phase.	Trials have shown that hypoglycemia triggers physiological changes promoting cardiac disease.
14.	Chiuve et al. [27].		2011	Cohort study.	81,722 females	Approximate the effects of a healthy lifestyle in decreasing sudden cardiac death in females.	Women that had multiple low-risk factors were less likely to die from sudden cardiac etiology.	Compliance with a good lifestyle in women confers a low risk for sudden cardiac arrest.
15.	Barthel et al. [28].		2011	Cohort study.	481	Mortality risk in diabetic patients post-myocardial infarction vs non-diabetics.	SCD is highly associated with abnormal autonomic function.	The prognosis of patients post-myocardial infarction is dim in patients with autonomic neuropathies.

## Review

Discussion

Type 2 diabetes mellitus (T2DM) is a chronic inflammatory disease that has a myriad of clinical implications in the body. The most prominent and studied outcome is macrovascular and microvascular diseases. There are clinical trials and cohort studies that show increased cardiovascular death in patients with type 2 diabetes.

Pathogenesis

**Figure 2:**
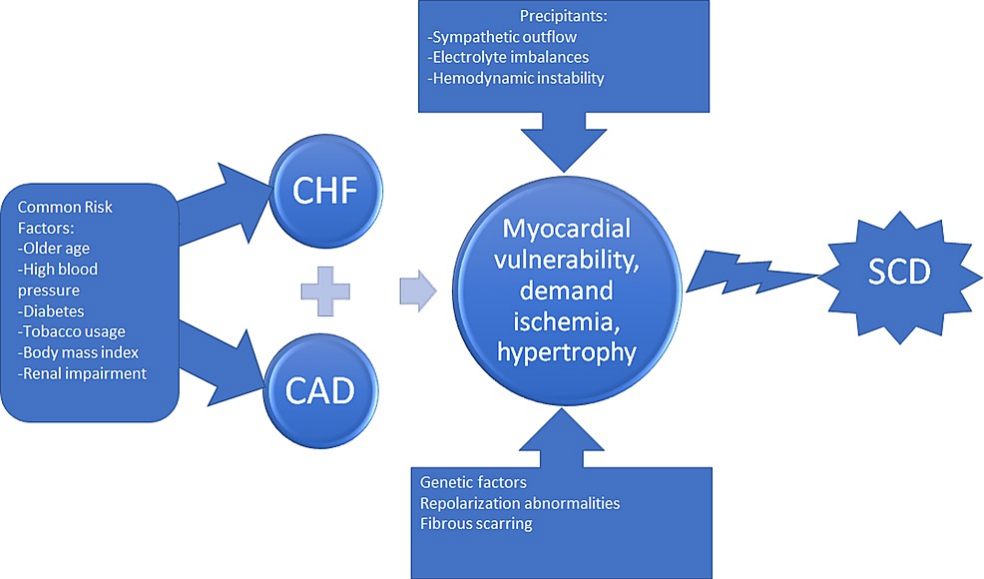
Pathogenesis of Sudden Death in Type 2 Diabetics. CHF-Congestive Heart Failure. CAD-Coronary Artery Disease. SCD-Sudden Cardiac Death.

Traditionally sudden cardiac death (SCD), as a group agreement by the American College of Cardiology/American Heart Association and World Health Organization, is defined as abrupt fatality within one hour since symptoms began or within 24 hours of being alive without symptoms [29]. The sudden arrest of cardiac activity has intricate pathophysiology, but several underlying factors affecting the heart are known. One study proposed that myocardial injury in the outpatient setting predisposes individuals to fibrotic changes of the cardiac muscle leading to aberrant conduction of electrical impulses around the fibrosis [30]. The mechanism of myocardial fibrosis is multifactorial but can be related to aging and coronary artery disease. The abnormal conduction from the fibrotic tissue leads to a collapse of circulation and decreased cardiac output because of a malignant arrhythmia. Another study focused on the mortality rates in patients with high sensitivity Troponin T (hsTnT) and deaths [30]. This study found a higher incidence of cardiac deaths in patients with increased levels of hsTnT and portends sudden death decades later. In the ambulatory setting, the high hsTnT led to a progressive decline in cardiac function over time that predisposed patients to cardiac death. Figure 2 below illustrates contributing factors to SCD.

The chain reaction of inflammation promotes oxidants such as reactive oxygen species that lead to the formation of atherosclerosis. The process of atherosclerotic plaque has to do with inflammatory cells and also oxidization of 'bad' cholesterol called LDL. Atheromas and atherosclerotic lesions are chronic intimal lesions of the vasculature. T2DM is a nidus for pro-oxidants and is associated with increased cholesterol levels in the serum. Increased intimal oxidized cholesterol is the key component of the atherosclerotic lesion as well as the cascade of events that follow inflammatory cells. An important sequela of inflammatory cells is the activation of the complement system. One prospective cohort in Asians demonstrated that Mannose-Binding Lectin (MBL) heralds a high-risk cardiovascular outcome [31]. In particular, the variant alleles (O/O genotype) had the highest hazard ratio for portending adverse cardiac outcomes (hazard ratio [HR] 3.43, 95% CI 1.24-9.49, p-value = <0.005). Additionally, a higher urine albumin/creatinine ratio led to poor outcomes (HR 1.58, 95% CI 1.0-2.48).

More commonly, sudden cardiac death in diabetics results from acute myocardial infarction. Post-mortem analysis of sudden cardiac death patients demonstrated approximately half of the deceased had chronic atherosclerotic plaques and fibrous cap instability [2]. The other half are theorized to have lethal ventricular electrical events from underlying heart disease. The mechanism of death is underlying ventricular fibrillation that precipitously decreases stroke volume and leads to pulselessness in a matter of minutes. In patients that were assessed before collapse and pulselessness, the most common documented arrhythmia originated within the ventricles. A cohort analysis of patients for 20 years demonstrated that ~90% of men and women had sudden death due to arrhythmia within the first hour [2].

Diabetic microvascular disease inflicts damage to the kidneys, retina, and peripheral nerves. Nephropathy in T2DM has traditionally been associated with cardiovascular complications such as ischemic heart disease, coronary artery disease, and myocardial infarction. T2DM has long been known as one of the most common risk factors for end-stage renal disease (ESRD) by non-enzymatic glycosylation of proteins in the arterioles of the glomerulus. The mechanism of coronary heart disease in chronic kidney disease is due to the cytokines and prolonged duration of reactive oxygen species that potentially contribute to the formation of atherosclerosis. Another proposed mechanism is the persistently elevated blood urea nitrogen (BUN) levels in diabetics that leads to proteins alteration [32]. Urea, made in the liver, chemically alters albumin by the process called carbamylation generating C-albumin. This process is hastened in kidney disease because the lack of amino acids promotes carbamylation of serum albumin. One treatment in patients with stage five chronic kidney disease (CKD) is the use of hemodialysis, in which blood is processed and filtered with a dialyzer. Large proteins are also filtered and trapped in the dialyzer that subsequently promotes further carbamylation of albumin. A randomized controlled trial called The Die Deutsche Diabetes Dialyse Studie (4D study) showed the effects of hydroxy methylglutaryl coenzyme A (HMG CoA) reductase inhibitors on survival during a four-year follow-up period in type 2 diabetics [32]. The study elucidates the significance of carbamylated albumin and the history of cardiac insufficiency and electrical conduction abnormalities. High levels of C-albumin had a one-year sudden death hazard ratio risk of 3.78. On the other hand, patients treated with statins showed statistically significant improvements in C-albumin levels as well as lower rates of sudden cardiac death and improved conduction in diabetic patients with ESRD.

Furthermore, Drechsler et al. studied the heart in dialyzed patients using ultrasound and demonstrated multiple associations with sudden cardiac arrest. Of note, patients with chronic kidney disease have a higher blood urea level that strains the heart to undergo hypertrophic changes and vascular remodeling [32]. In chronically elevated urea levels, patients are more likely to experience abnormalities in the conduction of electrical impulses from the sinoatrial node onto the hypertrophied cardiomyocytes [10]. In hemodialyzed patients, an increased thickness of the left ventricle is a poor harbinger of adverse cardiac outcomes. Radiologically, echocardiograms in patients with CKD had a positive correlation between heart failure with reduced ejection fracture and sudden cardiac death in patients with hypertrophic myocytes. Clinically speaking, when demographics are excluded, T2DM is an independent variable for sudden cardiac arrest [10]. The hypothesis is multifactorial but relates to stenotic coronary artery disease, prolonged QT interval, and small vessel vasculopathy causing neuropathies. A diabetic with an HbA1c of >8% was associated with an increased risk of sudden myocardial death when compared to a non-diabetic with an HbA1c of <6% [10].

Marcsa et al. studied five participants that experienced SCD were assessed for predisposing genes. The genes with the highest risk of SCD were pro-arrhythmic genes like the sodium voltage-gated channel alpha subunit five (*SCN5A*), ryanodine receptor two (*RYR2*), and nitric oxide synthase one adaptor protein (NOS1AP), transforming growth factor-beta receptor two (*TGFBR2*), and high adrenergic output genes. The *SCN5A* gene encodes for sodium channel receptors on the cardiomyocytes and activation of these channels elicits depolarization across the cell membrane. Defect in the *SCN5A* gene predisposes patients to a higher risk for lethal arrhythmias like prolonged QT and ventricular fibrillation [33]. The same study demonstrated microvascular outcomes were associated with a prolongation of the QT interval [33]. This is in concordance with a genetic predisposition in diabetes that leads to SCD.

Insults that cause myocardial stress eventually lead to a common pathway of hypertrophy, cardiac remodeling, and increased fibrogenesis through intracellular pathways [33]. A predominant pathway is the transforming growth factor-beta (TGFB) signaling cascade, which has been implemented in the development of sudden myocardial arrest in a population of coronary heart disease. In diabetic patients, ischemia to the myocardium and increased oxygen requirement by cardiomyocytes triggers an insidious cardiac remodeling and activation of the TGFB pathway. Another diabetic corollary of sudden death is mutations in the beta two adrenoreceptors found on cell membranes [33]. When found in susceptible obese individuals, this receptor mediates a sympathetic response in the body and acts as a trigger for sudden cardiac death.

Risk Factors

**Figure 3:**
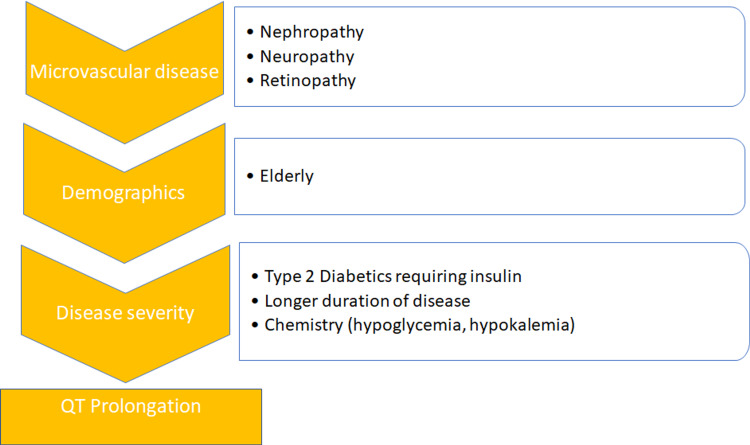
Substrates associated with a prolonged QT interval of >0.44 seconds.

Studies conducted on T2DM showed that QT prolongation is independently associated with high cardiovascular deaths in this patient population [20,29]. Considering a QTc cut-off of 0.44 seconds (secs), insulin therapy in type 2 diabetics is known for prolonging the QTc interval (95% CI <0.0001) [20]. In addition, severe microvascular complications such as nephropathy, retinopathy, and neuropathy were statistically significant risk factors for a prolonged QTc. Lastly, overweight elderly females with a diabetic prevalence of more than five years also showed the statistical and clinical significance of prolonged QTc [20]. Electrolytes such as potassium and calcium are known to affect the QT interval. Diabetic patients have lower total body potassium stores, especially under stress. Insulin stimulates the Na/K adenosine triphosphatase (ATPase) pump and causes an influx of two potassium cations in exchange for three sodium cations. Excess insulin therapy leads to relative hypokalemia and may precipitate torsades de pointes by prolonging the QT interval. Studies also show that patients on medications that block the formation or actions of angiotensin two (AT2) have a decreased QT interval [20]. Angiotensin-converting enzyme (ACE) inhibitors are commonly prescribed antihypertensives in T2DM because of elevated blood pressures or microalbuminuria. A list of potential factors correlated with an increased QT interval is illustrated in Figure 3 below.

Autonomic polyneuropathy is a major cause of significant lifestyle deterioration and future mortality in diabetic patients. The infamous “silent infarction” is related to autonomic dysfunction that causes an undetected myocardial infarction. Barthel et al. described patients that had severe autonomic dysregulation had the highest hazard ratios for cardiovascular mortality and sudden cardiac death (HR 4.9, 95% CI 2.4-9.9, P < 0.0001) [28]. During the post-infarction period, patients with severe dysfunctions in their autonomic nervous systems had higher risks of SCD [28].

**Figure 4:**
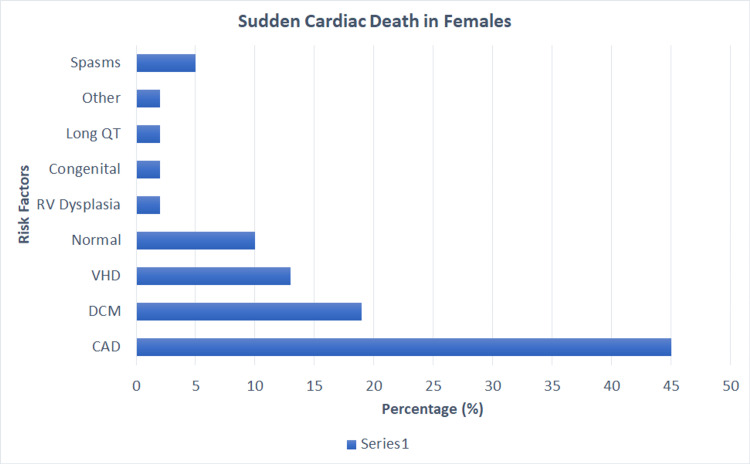
Risk factors attributing to sudden death in females from the AHA. CAD-Coronary Artery Disease. DCM-Dilated Cardiomyopathy. RV-Right Ventricular. VHD-Valvular Heart Disease. AHA-American Heart Association From Deo and Albert Study [2]

**Figure 5:**
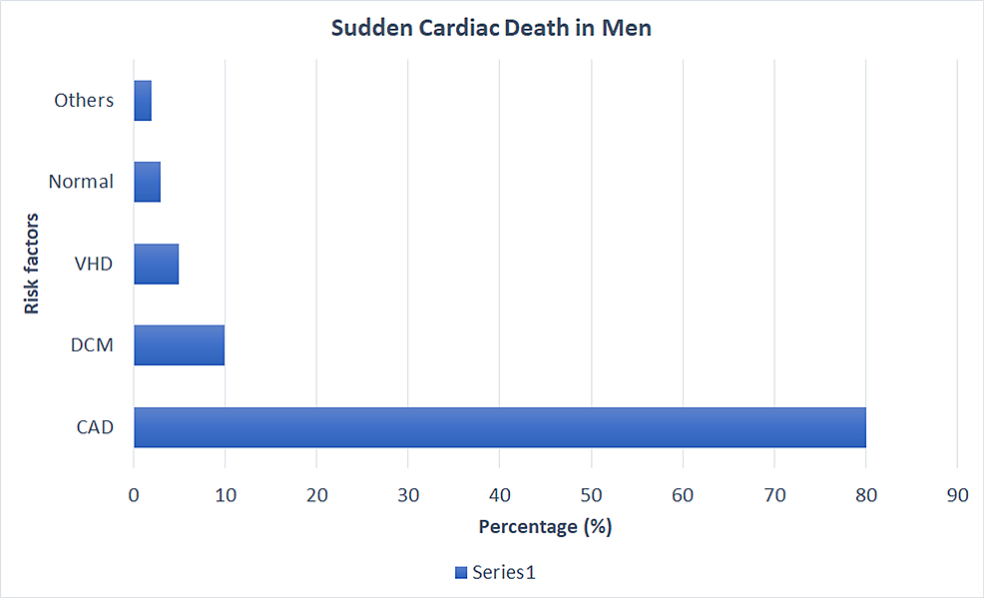
Risk factors attributing to sudden death in males from the AHA. CAD-Coronary Artery Disease. DCM- Dilated Cardiomyopathy. VHD-Valvular Heart Disease. AHA-American Heart Association. From Deo and Albert Study [2]

Eranti et al. expressed a correlation with increased deaths among diabetic males when compared to diabetic females [7]. Patients with a diabetogenic profile had a higher risk of death due to direct and independent associations with other comorbidities such as hypertension, dyslipidemia, and metabolic syndrome [7]. The study showed that patients with an impaired glucose tolerance at a cutoff of 172.50 mg/dL had a high accuracy as per the receiver operator curve for predicting SCD. This justifies the hypothesis of prediabetics and accelerated coronary artery intimal lesions. In addition, analyzing impaired glucose tolerance for 23 years showed the correlation of sudden cardiac death in patients with worsening one-hour oral glucose load [7]. Below are the summarized risk factors for females and males highlighted in Figure 4 and Figure 5, respectively [2].

Prevention

Most of the cardiac deaths occurring in females go unseen because the death precedes the clinical diagnosis of the underlying disease. The implantation of cardiac defibrillation devices serves to prevent death in patients with an ejection fraction of <30%. However, prevention aimed towards the risk factors for SCD is associated with an overall better prognosis in the future.

The factors that can be modified are associated with lifestyle changes such as tobacco consumption, body fat, sedentary activities, and dietary factors [5]. A Nurses’ Health Study showed that females with combined low-risk lifestyles such as constant exercise, balanced diet, non-smoking habit, and a healthy body mass index (BMI) were associated with lower cardiac deaths. Statistically, these women had a 92% chance of not dying from SCD when compared to women without these factors [27]. Moreover, obese patients that effectively obtained a BMI <25 reduced their risk for a prolonged QT interval [20].

Diabetes is a coronary artery disease equivalent to and has synergistic effects with hypertension and hyperlipidemia. Long-term effects of type 2 diabetes commonly lead to congestive heart failure and coronary heart disease that predispose to SCD. The associated comorbid conditions should be controlled with weight loss, diet, tobacco cessation, and exercise. The Mediterranean diet has a higher negative correlation with cardiac deaths when compared to other cardiac diets and consists of fish, vegetables, nuts, unsaturated fats, mild-moderate alcohol intake, and whole grains [27,34].

Friedman et al. studied dialyzed patients treated with omega-three polyunsaturated fatty acids and documented the outcomes. Although there is no definite research for assessing the effects of these fatty acids on cardiac death in dialyzed patients, there is a general inverse relationship. Serum omega-three fatty acids levels invariably demonstrated a reciprocal relationship with sudden myocardial death and mortality in type 2 diabetics [35]. In dialyzed patients, uremia and toxic waste products accumulated with defective native kidney function leading to systemic inflammation and diffuse pro-oxidant effects [32]. It is described that omega-three fatty acids counteract the harmful effects of chronic kidney disease by various mechanisms.

The sodium-glucose-like transporters type two (SGLT2) on the proximal convoluted tubules have significant clinical advantages in T2DM. The effects are contributed to increased glucose excretion by inhibition of these transporters and subsequently decreasing blood glucose levels. Fitchett et al. conducted a trial to show the benefits of empagliflozin on cardiovascular mortality in patients with coronary heart disease. Their study demonstrated the widespread efficiency and reduction in mortality throughout type 2 diabetics for all cardiovascular outcomes [18]. Empagliflozin is indicated in diabetics as adjunctive therapy in patients on oral metformin or with severe proteinuria.

ICDs are utilized for primary or secondary prevention of sudden cardiac death. Most commonly, ICDs are placed in patients with reduced ejection fractures or prior arrhythmias that can precipitate sudden death, such as ventricular fibrillation or unstable ventricular tachycardia. Recipients that are diabetics have perpetually higher mortality and morbidity when an underlying heart failure is present. Interestingly, type 2 diabetics that received the implant were at risk for sudden cardiac death. Patients with ICDs have had prolonged exposure to chronic inflammation with a preponderance of atherosclerotic changes and irreversible calcifications. These devices only prevent death from an electrical cause, but not the underlying cardiovascular comorbidities [36].

Limitations

This systematic review focuses on type 2 diabetes and the predictors of cardiac death. The limiters excluded patients with type 1 diabetes who suddenly died. Additionally, type 2 diabetes is a disease in late adulthood and, age is set to above 45 years old, excluded all studies below 45 years of age. Of the 15 articles included in the study, only two are randomized control clinical trials, and the hypothesis is based on the majority of observational literature. Additionally, patients with type 2 diabetes who died from non-cardiac causes such as cerebrovascular accidents were excluded. Lastly, analysis and assessment of sudden cardiac death are cumbersome. Autopsy reports can be inconclusive at times, but more importantly, families may opt out of investigating underlying etiologies due to personal believes.

## Conclusions

In conclusion, type 2 diabetes is related to sudden cardiac death due to various pathological reasons. The widespread atherosclerotic cardiovascular disease places subjects at high risks for myocardial infarction and progressively to arrhythmias. Ventricular fibrillation is the most lethal arrhythmia and has the highest risk in poorly controlled chronic diabetics, the elderly, people receiving insulin therapy, and people with severe microvascular complications. To prevent death from cardiac causes, diabetic patients must adhere to selective diets and treat underlying comorbidities. In the case of underlying myocardial dysfunction, implantable cardioverter-defibrillation devices are embedded; however, they do not improve the overall mortality and course in type 2 diabetes. This paper had several limitations as per the strict inclusion and exclusion criteria that may undermine the significance of future sudden deaths in type 2 diabetes mellitus. The disease prevalence is increasing worldwide because of advanced pharmacotherapy and can be preventable early on by addressing risk factors. In the future, literature should cultivate to include a preventive cause of sudden cardiac death and finding underlying etiologies before mortality. This paper encourages researchers to study the unknown causes of cardiac deaths in diabetes.
